# RAD51 Inhibition Induces R-Loop Formation in Early G1 Phase of the Cell Cycle

**DOI:** 10.3390/ijms22073740

**Published:** 2021-04-03

**Authors:** Zuzana Nascakova, Barbora Boleslavska, Vaclav Urban, Anna Oravetzova, Edita Vlachova, Pavel Janscak, Jana Dobrovolna

**Affiliations:** 1 Institute of Molecular Genetics of the Czech Academy of Sciences, 14220 Prague, Czech Republic; zuzana.nascakova@img.cas.cz (Z.N.); barbora.boleslavska@img.cas.cz (B.B.); VasekU@seznam.cz (V.U.); anna.oravetzova@img.cas.cz (A.O.); krizovaedita@seznam.cz (E.V.); 2 Faculty of Science, Charles University in Prague, 12800 Prague, Czech Republic; 3 Institute of Molecular Cancer Research, University of Zurich, 8057 Zurich, Switzerland

**Keywords:** RAD51, R-loop, B02 inhibitor, G1 phase of the cell cycle, origin of replication, pre-replication complex

## Abstract

R-loops are three-stranded structures generated by annealing of nascent transcripts to the template DNA strand, leaving the non-template DNA strand exposed as a single-stranded loop. Although R-loops play important roles in physiological processes such as regulation of gene expression, mitochondrial DNA replication, or immunoglobulin class switch recombination, dysregulation of the R-loop metabolism poses a threat to the stability of the genome. A previous study in yeast has shown that the homologous recombination machinery contributes to the formation of R-loops and associated chromosome instability. On the contrary, here, we demonstrate that depletion of the key homologous recombination factor, RAD51, as well as RAD51 inhibition by the B02 inhibitor did not prevent R-loop formation induced by the inhibition of spliceosome assembly in human cells. However, we noticed that treatment of cells with B02 resulted in RAD51-dependent accumulation of R-loops in an early G1 phase of the cell cycle accompanied by a decrease in the levels of chromatin-bound ORC2 protein, a component of the pre-replication complex, and an increase in DNA synthesis. Our results suggest that B02-induced R-loops might cause a premature origin firing.

## 1. Introduction

When nascent RNA invades the DNA helix and anneals to the template DNA strand, a three-stranded non-B DNA structure, called R-loop, is formed, with the non-template DNA strand exposed as a single-stranded DNA loop [1]. R-loops are formed co-transcriptionally behind the transcription complex. However, it has been reported that R-loops can be also formed *in trans* [2].

Over the past decade, several studies provided evidence that R-loops participate in normal cell physiology, such as “programmed” R-loop formation during class switch recombination, telomere maintenance, and mitochondrial DNA replication (reviewed in [3,4]). In recent years, there has been an increasing interest in the in vivo regulatory role of R-loop, such as their roles in transcription regulation. R-loops were found to be present abundantly in human gene promotors and terminators [5,6,7]. R-loop formation in CpG-rich domains promotes gene expression by preventing CpG methylation, an epigenetic modification associated with transcription silencing [5,8]. Additionally, promoter-proximal R-loops were found to facilitate the binding of transcription factors necessary for transcription initiation [9]. R-loops are also associated with the regulation of transcription termination by induction of repressive chromatin marks over gene terminators, thereby reinforcing RNA polymerase II (RNAPII) pausing at the polyadenylation signal [10].

In addition to physiologically occurring R-loops, perturbations in various cellular processes and a failure to control R-loop levels result in the accumulation of “aberrant” R-loops in the genome. The biological consequences of such R-loops include replication stress and DNA breakage, subsequently compromising genomic stability, which may lead to human disease [11,12,13]. Given this negative impact of R-loops, their formation and resolution must be strictly regulated and identification of the factors that are involved in R-loop sensing, signaling, and resolving is of great interest.

While a number of factors has been shown to prevent R-loop formation, e.g., mRNA processing factors [14,15], and to promote R-loop resolution, e.g., RNase H1 [14,16,17] or Senataxin [18], only very little is known about the factors promoting the formation of R-loops. Bacterial RecA, a strand exchange protein that promotes invasion of single-stranded DNA into duplex DNA during homologous recombination, and its eukaryotic homolog, Rad51, have been shown to promote RNA:DNA hybrid formation in vitro [7,19,20]. Wahba et al. reported that, in yeast *Saccharomyces cerevisiae*, R-loop formation and the associated genome instability require Rad51 [7]. Thus, we sought to explore whether this protein has the potential to promote hybrid formation in human cells. Given the essential role of human RAD51 protein in DNA damage response, the progression of RAD51-deficient cells through the cell cycle is strongly impaired; therefore, we inhibited its activity chemically using a RAD51 inhibitor called B02 [21]. Although the exact mechanism of B02 is not known, it was repeatedly shown to have a biological effect in human cells [22,23]. We demonstrated that B02 disrupts the formation of RAD51 foci in cell nuclei [24].

Most research on R-loops was carried out using the so-called S9.6 antibody, which displays an affinity for RNA:DNA hybrids [25]. Another approach relies on a cellular system with an inducible expression of a catalytically inactive RNase H1 fused with green fluorescent protein (RNH1(D210N)-GFP). This mutant form of RNase H1 can recognize and bind to R-loops but, due to an inactivating mutation in the nuclease catalytic site, is not able to cleave the RNA moiety within the hybrid, thus increasing R-loop stability [26,27,28]. We took advantage of this binding of the RNase H1 nuclease-deficient variant to RNA:DNA hybrids to gain a better understanding of molecular mechanisms involved in the formation of R-loops and subsequent genesis of genomic instability. Surprisingly, we found that inhibition of the RAD51 activity by B02 as well as small interfering RNA (siRNA)-mediated depletion of RAD51 did not prevent R-loop formation induced by diospyrin D1, an inhibitor of spliceosome assembly. Interestingly, we observed that the treatment of human cells with B02 resulted in a RAD51-dependent accumulation of R-loops in the early G1 phase of the cell cycle. Our data suggest that these R-loops might cause premature initiation of DNA synthesis in the early G1 phase.

## 2. Results and Discussion

### 2.1. RAD51 Inhibition Induces the Formation of R-Loops

**Figure 1:**
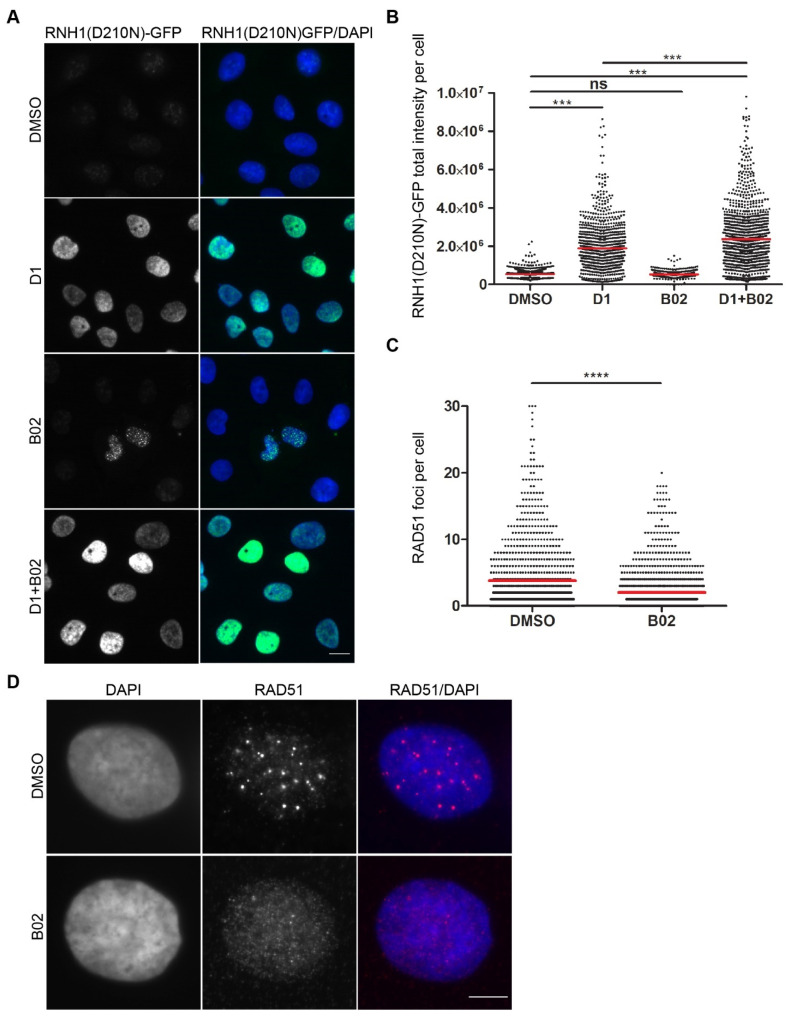
RAD51 is not required for R-loop formation induced by the inhibition of spliceosome assembly. (**A**,**B**) U-2-OS T-REx (RNH1(D210N)-GFP) cells were treated with doxycycline (1 ng/mL) for 24 h. B02, a RAD51 inhibitor, (20 µM) was added for the last 6 h during doxycycline treatment. R-loop formation was induced by diospyrin D1 (20 µM), an inhibitor of spliceosome assembly. Pre-extracted and fixed cells were counterstained with 4′6-Diamidine-2′-phenylindole (DAPI) and subjected to image-based analysis of the GFP signal. Representative images are shown in (**A**) alongside the quantification in (**B**). The data in (**B**) are pooled from 3 independent experiments. The red line represents the mean value. (**C**,**D**) Cells treated with B02 (20 µM) for 6 h were subjected to immunofluorescence staining of RAD51. The representative images shown in (**D**) alongside the quantification of RAD51 foci in an asynchronous population in (**C**). Scale bar in (**A**,**D**) represents 10 µm. Statistical significance was determined using the Unpaired *t* test (**** *p* < 0.0001, *** *p* < 0.005, ns, not significant).

RAD51 is an essential DNA repair factor in cells, and its absence is lethal [29]. It has been shown that its bacterial homolog RecA promotes RNA:DNA hybrid formation in vitro [19,20] and its yeast counterpart in vivo [7]. To investigate whether the RAD51 protein is actively involved in the formation of co-transcriptional R-loops in human cells, we took advantage of human U-2-OS T-REx cells with a stably integrated cassette for inducible expression of a catalytically inactive mutant of RNase H1 fused with green fluorescent protein (RNH1(D210N)-GFP) [26,27]. The formation of R-loops was induced by diospyrin D1, an inhibitor of spliceosome assembly [30,31]. We observed that diospyrin D1 induced the pan-nuclear accumulation of chromatin-bound RNH1(D210N)-GFP, indicating the presence of R-loops (Figure 1A,B). To test whether diospyrin D1-induced R-loop formation requires the activity of RAD51, we exposed the cells simultaneously to diospyrin D1 and the RAD51 inhibitor B02. We found that the chemical inhibition of RAD51 rather increased accumulation of chromatin-bound RNH1(D210N)-GFP) in B02-treated cells (Figure 1A,B), excluding a primary role of the homologous recombination machinery in promoting R-loop formation. Interestingly, we observed the accumulation of RNH1(D210N)-GFP foci in a small fraction of B02-treated cells without the addition of diospyrin D1 (Figure 1A and Figure S1), suggesting that B02 induces R-loop formation. As expected, the treatment of cells with B02 resulted in a strong reduction in the number of RAD51 nuclear foci (Figure 1C,D) [24].

### 2.2. B02-Induced R-Loops Occur in Early G1 Phase of the Cell Cycle

**Figure 2:**
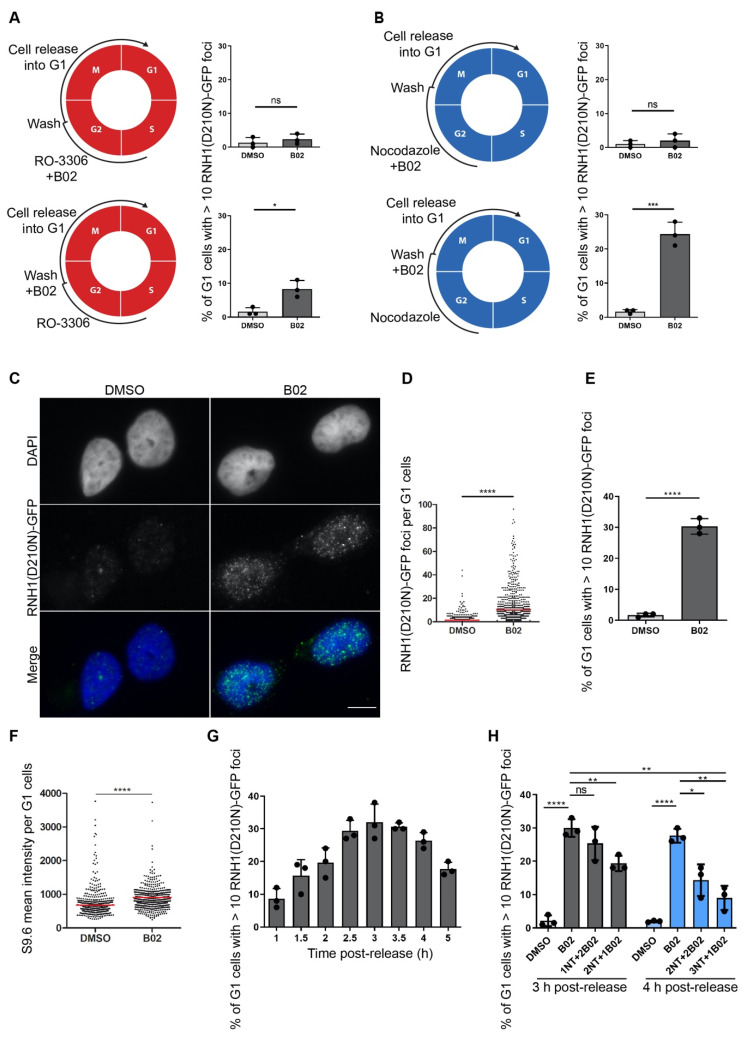
RAD51 inhibitor B02 induces the formation of R-loops in the early G1 phase of the cell cycle. (**A**,**B**) U-2-OS T-REx (RNH1(D210N)-GFP) cells were synchronized in the G2 or prometaphases with RO-3306 (9 µM, 16 h) and nocodazole (100 ng/mL, 20 h), respectively. B02 was added for the last 6 h of treatment with synchronization agents (top panel) or after the cells were released from cell cycle arrest (bottom panel) for 3 h (nocodazole) or 4 h (RO-3306). Doxycycline (1 ng/ mL) was present for 24 h. Schematic representations of the protocol alongside the quantification of fraction of G1 cells with more than 10 RNH1(D210N)-GFP foci are shown for RO-3306-treated cells in (**A**) and nocodazole-treated cells in (**B**). (**C–E**) B02 induces the accumulation of RNH1(D210N)-GFP foci in G1 cells. Cells expressing RNH1(D210N)-GFP were released from nocodazole-mediated cell cycle arrest (100 ng/mL nocodazole for 20 h) for 3 h and simultaneously treated with B02 (20 µM). Representative images of cells with RNH1(D210N)-GFP foci are shown in (**C**) alongside the image-based quantification of the number of RNH1-GFP foci per cell in (**D**) and the fraction of G1 cells with more than 10 RNH1(D210N)-GFP foci in (**E**). Scale bar represents 10 µm. (**F**) Effect of B02 on the formation of R-loops in G1 cells assessed by immunostaining with the S9.6 antibody, which recognizes RNA:DNA hybrids. Cells were treated as in (**C**). Image-based quantification of the nucleoplasmic S9.6 signal is shown. (**G**,**H**) B02-induced R-loops are formed in the early stages of the G1 phase and are resolved with increasing time. (**G**) Cells were released from the nocodazole-mediated block for the indicated time points and simultaneously treated with B02 (20 µM). A graph represents quantification of the fraction of the G1 cells with more than 10 RNH1-GFP foci per nucleus. (**H**) B02 (20 µM) was added into the cell culture medium 1, 2, or 3 h post-release from cell cycle arrest for a total time of 3 h or 4 h. The graph represents a quantification of the fraction of the G1 cells with more than 10 RNH1-GFP foci per nucleus (in %). The data shown in (**A**,**B**,**D**–**H**) are pooled from 3 independent experiments. Statistical significance was determined using Unpaired *t* test (**** *p* < 0.0001, *** *p* < 0.005, ** *p* < 0.01, * *p* < 0.05, ns, not significant).

We sought to explore whether B02-induced R-loops occur in a specific phase of the cell cycle. Based on our observation that RNH1(D210N)-GFP-positive cells were detected always in pairs in close proximity and on the software-based analysis of physical features of these cells, including the pan-nuclear intensity of the 4′6-Diamidine-2′-phenylindole (DAPI) signal and the area of the nuclei, we suspected that the R-loop-positive cells upon B02 treatment were present exclusively in the G1 phase of the cell cycle. However, the percentage of the cell population positive for the GFP marker was not sufficient for any further analysis. Therefore, we sought to investigate whether cell synchronization might lead to an increased number of GFP-positive cells in the cell population. We exposed the cells to two inhibitors of cell cycle progression: RO-3306 or nocodazole. RO-3306, a selective inhibitor of CDK1, reversibly arrests the proliferating cells at the G2/M transition point, with rapid entry into mitosis after its removal. RO-3306-mediated synchronization of the cell population was followed by release from cell cycle arrest until the cells reached the G1 phase. The cells were exposed to B02 during the time of release from G2 arrest. We found that RO-3306-mediated synchronization mildly increased the percentage of G1 cells positive for the GFP signal (Figure 2A, bottom panel). Nocodazole, a reversible inhibitor of microtubule polymerization, arrests the proliferating cells in mitosis at the prometaphase phase with a very rapid release from the block after its removal. The synchronization of cells in mitosis allowed us to isolate the mitotic cells, a method commonly referred to as a mitotic shake-off, and to discard the cells that were not yet arrested in the M phase. The nocodazole-synchronized cells were released from arrest into the G1 phase and simultaneously exposed to B02. We observed a massive increase in cells positive for the GFP signal within the G1 population (Figure 2B, bottom panel). Based on quantitative image-based cytometry (QIBC) data, 20–30% of the G1 population was GFP-positive (assessed as a cell with more than 10 GFP foci per nucleus) (Figure 2B bottom panel, Figure 2E). To investigate whether the origin of G1-specific R-loops is in the previous cell cycle, we exposed the cells to B02 simultaneously with RO-3306 and nocodazole treatment, respectively, followed by release from cell cycle arrest in the absence of B02 until the cells reached the G1 phase. We observed that the number of GFP-positive cells was lower than 5% (Figure 2A,B, upper panel), a level similar to an asynchronous population exposed to B02 (Figure S1). Additionally, immunostaining of the DNA damage markers, such as 53BP1 and phosphorylated histone H2AX (γH2AX), did not indicate the presence of DNA damage in B02-exposed cells, since the level of both DNA damage markers was comparable with dimethyl sulfoxide (DMSO)-treated cells (Figure S2). All together, these results exclude the possibility of G1-specific R-loop formation as a consequence of unresolved issues from the previous cell cycle.

The characteristic feature of B02-induced GFP-positive cells is a high number of RNH1(D210N)-GFP foci in a range from 10 to 100 foci per nucleus (Figure 2C,D). Immunostaining of B02-treated cells with the S9.6 antibody, which specifically recognizes RNA:DNA hybrids, confirmed the elevated level of R-loops upon B02 treatment in G1 cells (Figure 2F). We next asked whether the B02-induced R-loops are permanent or dynamic structures. To investigate the dynamics of R-loops, we synchronized the cells with nocodazole, released them from mitotic arrest, and harvested them at times from 1 to 5 h (indicated in Figure 2G). Using QIBC analysis, we observed that the highest number of cells present in the G1 phase was 3 to 4 h post-release and that the highest number of GFP-positive cells was 3 h post-release. The number of GFP-positive cells decreased with increasing time post-release (Figure 2G), suggesting that B02-induced R-loops are highly dynamic structures. We next wanted to address whether the G1-specific R-loops are formed as a consequence of B02-mediated RAD51 inhibition affecting any process in the mitosis or at the beginning of the G1 phase. To investigate this, we released the cells from pro-metaphase arrest, added B02 into the cell culture media at later time points (1, 2, or 3 h post-release), and harvested the cells 3 or 4 h post-release. Based on the QIBC data, we found that the addition of B02 after 1 or 2 h post-release, with a total time of release being 3 h, reduced the number of GFP-positive cells only mildly when compared to cells released directly to the medium containing B02 for 3 h (Figure 2H, gray bars). However, we observed a robust reduction in GFP-positive cells when we added B02 3 h post-release, for a total time of release of 4 h (compared to the 3 or 4 h lasting treatment with B02) (Figure 2H, blue bars). These results suggest that G1-specific R-loops form as a result of B02-mediated effects specific for an early G1 phase of the cell cycle. Taken together, our results suggest that B02-induced R-loops are highly dynamic structures formed specifically in the early G1 phase and presumably are resolved by the time cells enter the late G1 phase.

### 2.3. Formation of B02-Induced R-Loops Requires RAD51

**Figure 3:**
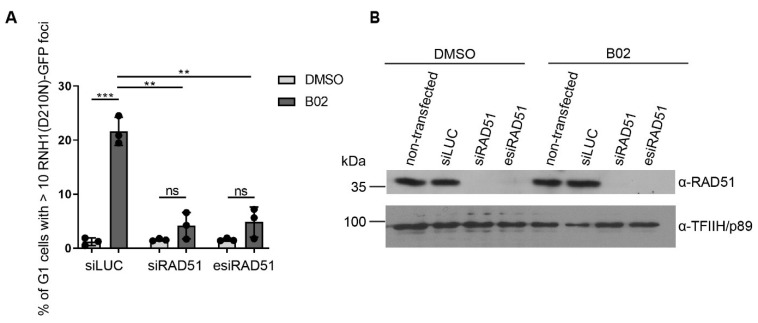
B02-induced R-loops are dependent on the presence of RAD51. (**A**,**B**) U-2-OS T-REx (RNH1(D210N)-GFP) cells were transfected with small interfering RNA (siRNA) against RAD51 (siRAD51) or a pool of endoribonuclease-prepared siRNAs (esiRNA) against RAD51 (esiRAD51) for 36 h. For the last 24 h, the cells were treated with doxycycline (1 ng/mL) and nocodazole (100 ng/mL), released from arrest for 3 h and pre-extracted before fixation. Cells were then subjected to image-based analysis of RNH1(D210N)-GFP foci. (**A**) The graph represents quantification of the fraction of G1 cells with more than 10 RNH1-GFP foci per nucleus (in %). (**B**) Protein levels were assessed by Western blot analysis. The data shown in (**A**) are pooled from two independent experiments. Statistical significance was determined using Unpaired *t* test (*** *p* < 0.005, ** *p* < 0.01, ns, not significant).

The biological effect of B02 was demonstrated in vitro and in various human and mouse cell lines [21,22,23,32,33]. B02 was reported to efficiently and specifically bind to RAD51 and to inhibit its DNA strand exchange activity in vitro [21]. However, the exact mechanism and possible off-targets of this inhibitor remains unknown. To test whether B02-mediated R-loop formation is RAD51-dependent, B02 was added to RAD51-depleted and nocodazole-synchronized cells. We found that knockdown of RAD51 strongly reduced the number of GFP-positive cells within the G1 cell population (Figure 3A,B). These results suggest that the formation of B02-induced G1-specific R-loops is dependent on the presence of RAD51. However, due to the little information about the mechanism of action of B02, we cannot rule out that a canonical activity mediated by RAD51, such as single-stranded DNA-binding or strand invasion, is not involved in the B02-induced G1-specific phenotype. Recently, RAD51 was shown to promote the formation of R-loop structures at telomeres. This study demonstrated that RAD51 physically interacts with telomeric-repeat-containing RNA (TERRA) in vitro, a type of long-noncoding RNA (lncRNA) transcribed from chromosome ends, and facilitates the formation of telomeric R-loops after transcription. RAD51-dependent telomeric R-loop formation is a novel mechanism for the recruitment of lncRNAs to new loci *in trans* [2].

### 2.4. Inhibition of Transcription Initiation Suppresses Formation of B02-Induced R-Loops

**Figure 4:**
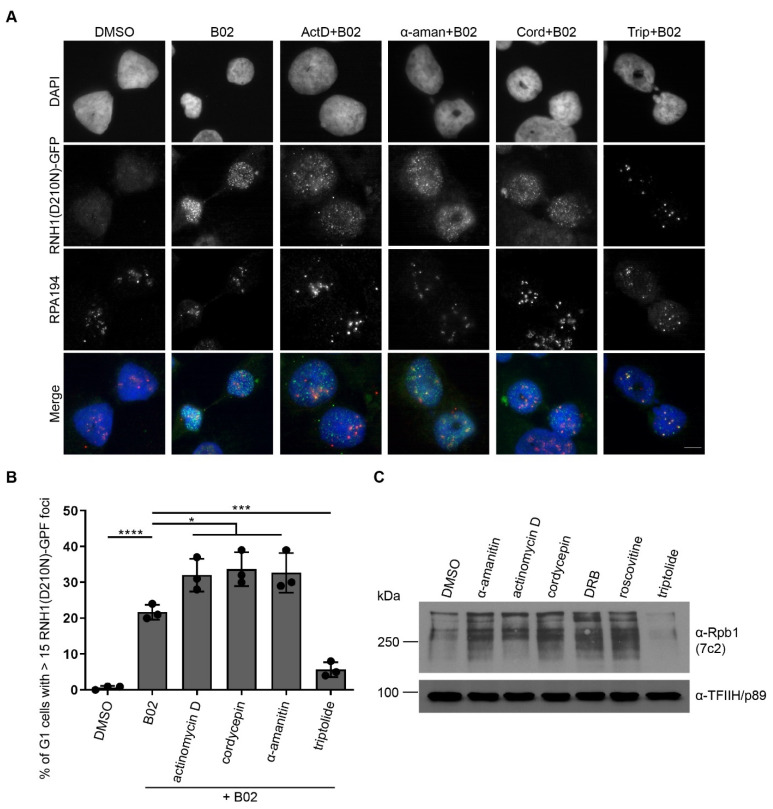
B02-induced R-loops are dependent on RNA polymerase II (RNAPII) transcription initiation but not elongation. (**A**–**C**) U-2-OS T-REx (RNH1(D210N)-GFP) cells were treated with doxycycline (1 ng/mL) for 24 h, simultaneously with nocodazole (100 ng/mL) for last 20 h and with B02 (20 µM) for 3 h post-release from the nocodazole-mediated block. Actinomycin D (1 µg/mL), α-amanitin (2 µg/mL), cordycepin (50 µM), triptolide (1 µM), 5,6-dichloro-1-β-D-ribofuranosyl-benzimidazole (DRB) (50 µM), or roscovitine (50 µM) were added simultaneously with B02 for 3 h. Pre-extracted and fixed cells were then subjected to immunostaining of the RNA polymerase I subunit, RPA 194, and DAPI staining. Representative images of RNH1(D210N)-GFP-positive cells are shown in (**A**) alongside the image-based quantification of the fraction of G1 cells with more than 10 RNH1-GFP foci per nucleus in (**B**). The data shown in (**B**) are pooled from 3 independent experiments. Statistical significance was determined using Unpaired *t* test (**** *p* < 0.0001, *** *p* < 0.005, * *p* < 0.05). (**C**) The protein levels of the chromatin-bound RNAPII subunit (RBP1) as well as TFIIH (p89) were assessed by Western blot analysis.

In the vast majority of R-loops formed in vivo, the RNA strand was generated de novo during ongoing transcription [34]. The co-transcriptional origin of R-loops was demonstrated by treatment of the cells with transcription inhibitor 5,6-dichloro-1-β-D-ribofuranosyl-benzimidazole (DRB), an inhibitor of CDK9 (the kinase of the positive transcription elongation factor b (P-TEFb)) that rapidly arrests transcription. The strong reduction in R-loops was observed in DRB-treated cells (measured by RNA:DNA hybrid immunoprecipitation followed by high-throughput sequencing). Additionally, removal of DRB led to a progressive reappearance of R-loops, demonstrating that co-transcriptional R-loop formation is a highly dynamic process [35]. To test whether B02-induced R-loops are transcription-dependent, we treated the cells with various transcription inhibitors simultaneously with B02 and analyzed the accumulation of RNH1(D210)-GFP foci in G1 cells. For these experiments, we selected the following transcription inhibitors: actinomycin D, a DNA intercalating agent blocking the RNA polymerase progression; α-amanitin, which block the transcription by preventing nucleotide incorporation and translocation of the transcript; cordycepin, a nucleoside adenosine derivative inhibiting transcription elongation; DRB, preventing the transcription elongation; and triptolide, an inhibitor of the helicase activity of XPB (the subunit of TFIIH), inhibiting RNAPII-dependent transcription by preventing the formation of “transcription bubble”. In agreement with the literature data, we observed that the effect of triptolide on transcription machinery is different compared to other transcription inhibitors. While most of the transcription inhibitors allow RNA polymerase recruitment to DNA and partial initiation of nascent pre-mRNA synthesis, triptolide blocks the RNAPII binding to DNA and induces a proteasome-dependent degradation of RNAPII, thereby inhibiting transcription at the initiation step. We found that binding of the largest subunit of the RNAPII complex (RPB1) to chromatin was completely abolished by triptolide (Figure 4C), compared to the other inhibitors, where RNAPII binding to DNA was not suppressed (Figure 4C). We found that inhibition of transcription by the aforementioned inhibitors except for triptolide did not prevent the formation of B02-induced R-loops (Figure 4A,B). Surprisingly, these inhibitors (except for triptolide) increased the number of GFP-positive cells within the G1 population compared to the B02 treatment only (Figure 4B). On the contrary, inhibition of the RNAPII-dependent transcription by triptolide suppressed the formation of G1-specific R-loops (Figure 4B). In triptolide-treated cells, co-staining of RPA 194, a subunit of the RNA polymerase I (RNAPI) complex and nucleolar marker, and R-loops showed colocalization of the remaining B02-induced R-loops and RPA 194 (Figure 4A). These results indicate that B02-induced R-loops resistant to triptolide are formed due to RNAPI-dependent transcription of ribosomal DNA (rDNA) genes, which is not affected by triptolide treatment.

Taken together, our data support the model wherein R-loops are formed co-transcriptionally. We showed that B02-induced G1-specific R-loop formation depends on transcription initiation. Surprisingly, we noticed elevated R-loop levels upon the treatment of cells with transcription elongation inhibitors, such as DRB, which is known to block RNAPII elongation beyond the major pausing site of RNAPII near the transcription start site (TSS). This prompted us to think that RNAPII binding to DNA, opening a “transcription bubble”, and initiation of transcription, which is then blocked by the effect of an inhibitor, make it possible to form R-loops behind the transcription complex. Our observations are supported by the literature data reporting that elevated pausing of RNAPII at TSS promotes R-loop formation [6]. However, upon triptolide treatment, the RNAPII biding to DNA is completely abolished, making it impossible for transcription to start, and thus, the R-loop level is strongly reduced.

### 2.5. B02-Induced R-Loops Form at Transcription Start Site of rDNA Locus

**Figure 5:**
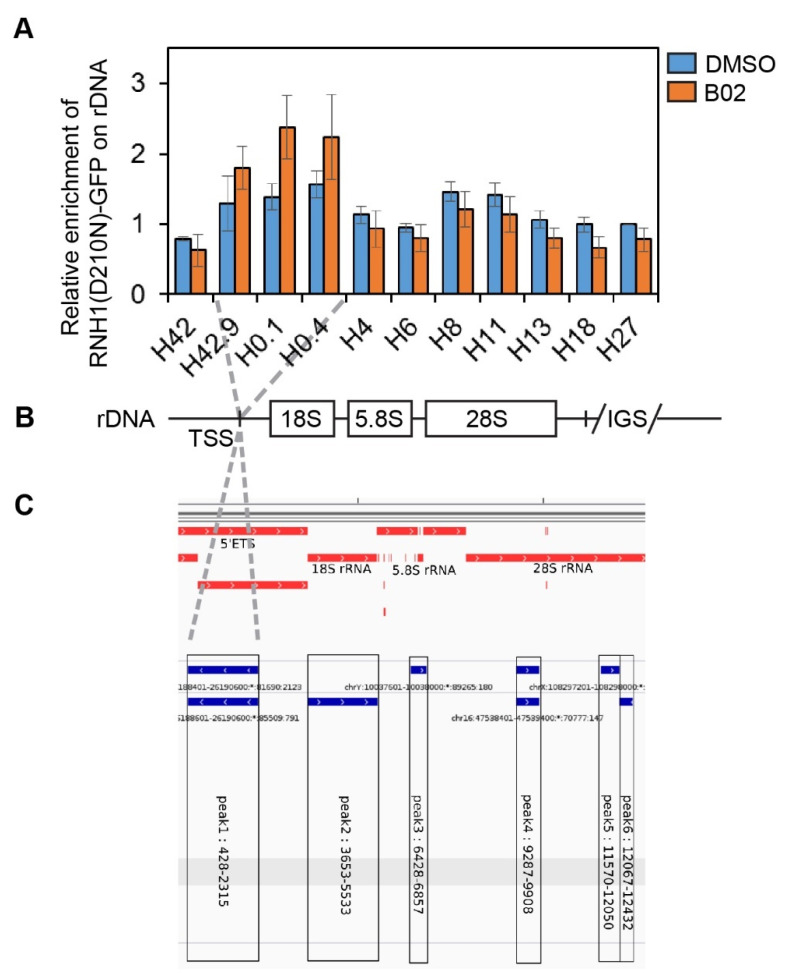
R-loops accumulate around the rDNA transcription start site upon B02 treatment. (**A**) U-2-OS T-REx (RNH1(D210N)-GFP) cells were treated with doxycycline (1 ng/mL) for 24 h simultaneously with nocodazole (100 ng/mL) for the last 20 h and with or without B02 (20 µM) for 3 h post-release from cell cycle arrest. After pre-extraction and fixation, the cells were subjected to chromatin immunoprecipitation of RNH1(D210N)-GFP-bound regions followed by quantitative PCR analysis (qPCR). The data were analyzed by taking the cycle threshold values from a qPCR assay. Relative enrichment was calculated and normalized to the amplicon H27 of non-treated sample. The data plotted are pooled from 3 experiments. (**B**) Schematic representation of primer alignment to the rDNA locus. (**C**) Print-screen from the Integrative Genomics Viewer (IGV) browser for human rDNA (acquired in July 2017) presenting localization of the replication origins within the ribosomal DNA locus (blue lines).

Genomic profiling of R-loops shows that a largest fraction was observed over gene bodies; however, a significant part was mapped to the promoter and terminator regions [6,35,36]. As expected, the promoters prone to forming R-loops were enriched for GC skews [8], which is consistent with a higher stability of R-loops carrying the G-rich RNA strand [37]. Further evidence suggests that co-transcriptional R-loop formation at promoter regions might play a functional role in protection against DNA methylation, indicating a role of R-loops in the regulation of gene expression [8]. We sought to explore the possibility that B02-induced R-loops form at the promoter regions. To investigate this, we mapped the occupancy of RNH1(D210N)-GFP along the rDNA locus, which is heavily transcribed by RNAPI and is known to be prone to forming R-loops (Figure 5A,B) [6]. We treated cells released from nocodazole-mediated cell cycle arrest with B02 or DMSO and performed chromatin immunoprecipitation of RNH1(D210N)-GFP-bound genomic loci followed by quantitative PCR analysis. We found that RNase H1 has preferential binding sites within the rDNA region (Figure 5A, DMSO). Surprisingly, B02 strongly enhanced the recruitment of RNH1(D210N)-GFP to the promoter-proximal region (≈ 400 bp) including the transcription start site (TSS) of rDNA repeat (Figure 5A, B02). These results are consistent with the previous observations that RNA:DNA hybrids accumulate over the RNAPI promoter and 5’-ETS regions of the rDNA in RNase H1-deficient yeast [38].

### 2.6. B02 Induces DNA Synthesis in Early G1 Phase

**Figure 6:**
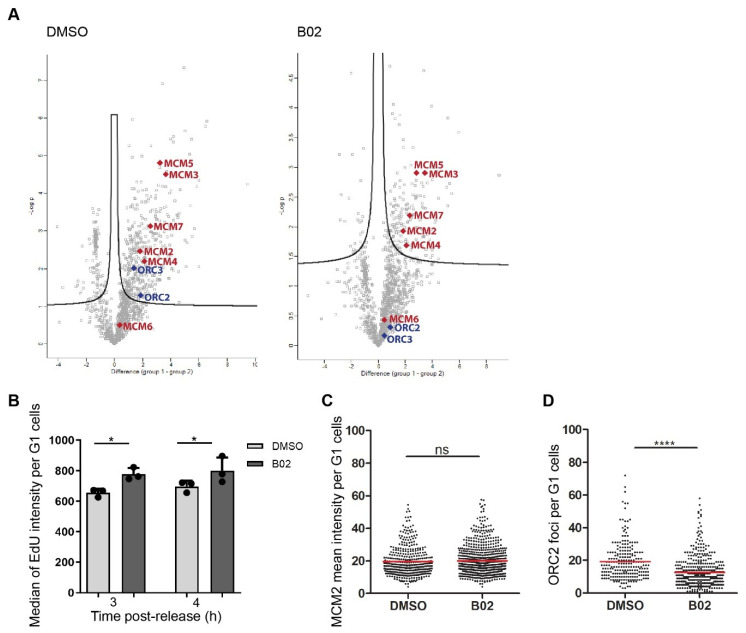
B02 induces DNA synthesis in the early G1 phase. (**A**) Mass-spectrometry screen of proteins associated with R-loops upon B02 treatment. Volcano plots for significantly abundant proteins identified in cells expressing RNH1(D210N)-BioID2-HA chimeric protein. Comparison of cells expressing the fusion protein treated (right panel) or non-treated (left panel) with B02 (20 µM) with the cells that were treated with the vehicle alone (dimethyl sulfoxide, DMSO). Each plot represents the difference in expression of particular proteins between the two conditions plotted against the level of statistical significance (false discovery rate (FDR) 0.2). Significantly abundant proteins are in the upper right corner of the volcano plots. Cell were treated with doxycycline for 24 h, and nocodazole was added for the last 20 h. After release from cell cycle arrest, DMSO or B02 (20 µM), and biotin (20 µM) were added for 4 h. The origin licensing proteins are highlighted on the volcano plots, namely Origin Recognition Complex (ORC, blue) and Minichromosome Maintenance Complex (MCM, red). (**B**) Analysis of 5-Ethynyl-2′-deoxyuridine (EdU) incorporation by flow cytometry. U-2-OS cells were treated with B02 (20 µM) and EdU (20 µM) for 3 h or 4 h post-release from nocodazole-induced cell cycle arrest, pre-extracted, fixed, and subjected to flow cytometry analysis. The data on the graph represent median values per condition from 3 independent experiments. (**C**) Image-based quantification of nuclear signal of MCM2 in G1 cells. (**D**) The image-based quantification of number of ORC2 foci in G1 cells. The data shown in (**C**–**D**) are pooled from 3 independent experiments. The red lines represent the mean values. Statistical significance was determined using Unpaired *t* test (**** *p* < 0.0001, * *p* < 0.05, ns, not significant).

We next set out to identify the proteins interacting with B02-induced G1-specific R-loops. We took advantage of the promiscuous variant of biotin ligase, which, upon the addition of external biotin, is able to add a biotin tag to proteins in its close proximity. We established a U-2-OS-based cell line with a stably integrated cassette inducibly expressing the catalytically inactive RNase H1 fused with biotin ligase (BioID2) and human influenza virus hemagglutinin (HA) tag. The cells were synchronized with nocodazole, and non-mitotic cells were discarded from further analysis via mitotic shake off. Nocodazole-synchronized mitotic cells were released from cell cycle arrest and treated with B02 or DMSO during the time of release, followed by protein immunoprecipitation of biotinylated proteins and subsequent mass spectrometry-based proteomic profiling. It was previously reported that promoter regions are hotspots for R-loop formation as well as preferential sites for the initiation of replication [6,39]. Additionally, we found that RNH1 preferentially binds to TSS within the rDNA locus upon B02 treatment (Figure 5A,B). Within the same region, two replication origins were identified (Figure 5C). Therefore, we sought to investigate whether proteins of replication origin licensing would be identified in our proteomic profiling. As we expected, the proteins of origin recognition complex (ORCs) and minichromosome maintenance complex (MCMs) were significantly abundant in DMSO-treated compared to unsynchronized cells (Figure 6A, DMSO). Surprisingly, a comparison of the protein profiling of DMSO- and B02-treated cells to parental U-2-OS cells showed that B02 treatment reduced the abundance of ORC proteins while the proteins of the MCM complex remained significantly abundant (Figure 6A, B02), suggesting that B02 might alter the process of replication origin licensing or firing.

Based on a model of replication origin licensing, the sites of origin of DNA replication are marked by the ORC protein complex, for which the assembly on DNA starts during late mitosis. Through the G1 phase, additional factors are recruited to the ORC-licensed origins, including MCM helicase complex (MCM2-7). Soon after the MCM complex is loaded onto the origin DNA, the ORC complex dissociates and, upon recruitment of additional factors, the pre-replication complex is assembled and prepared to be fired in the following S phase [40,41,42,43,44]. We suspected that licensing of replication origins is dysregulated upon B02 treatment; therefore, we next set out to investigate whether B02 interferes with a proper replication origin licensing and firing. To monitor the progression of fired origins, cells released from cell cycle arrest, were pulse-labeled with 5-Ethynyl-2’-deoxyuridine (EdU) for the total time of release, and simultaneously treated with B02. The EdU intensity was measured by flow cytometry. We found that the EdU signal was significantly increased in B02-treated cells compared to DMSO-treated cells harvested 3 h post-release (Figure 6B), with the difference being slightly more profound in cells harvested 4 h post-release (Figure 6B). EdU incorporation might occur due to unscheduled DNA repair synthesis following the processing of R-loops by nucleotide excision repair (NER) endonucleases [45]. Increased levels of NER-dependent H2AX phosphorylation was previously reported in G1 cells in response to UV irradiation [46]. However, we did not observe increased levels of DNA damage markers in B02-treated cells in comparison to DMSO-treated cells (Figure S2), suggesting that B02-induced EdU incorporation is not a result of DNA repair synthesis. Finally, QIBC analysis revealed a reduced number ORC2 nuclear foci in B02-treated cells compared to control cells (Figure 6D and Figure S3B), while the pan-nuclear signal of MCM2 was not altered by B02 treatment (Figure 6C and Figure S3A). Taken together, our results suggest that ORCs might dissociate from replication origins upon B02 treatment, leading to a premature induction of origin firing.

## 3. Experimental Procedures

### 3.1. Cell Culture and Chemicals

U-2-OS (ATCC HTB96, a human osteosarcoma cell line) T-REx cell lines carrying cassettes for the expression of catalytically inactive RNase H1 tagged with green fluorescent protein (RNH1(D210N)-GFP) (the cell line described in [27]) or with a biotin ligase (BioID2) and an HA epitope tag (RNH1(D210N)-BioID2-HA) were cultivated in Dulbecco’s modified Eagle’s medium (DMEM, Gibco) supplemented with 10% fetal bovine serum (FBS, Tet-free approved, Gibco), 100 U/mL penicillin, and 100 μg/mL streptomycin. Cells stably transfected with pAIO plasmids carrying GFP and BioID-fusion constructs were selected in the presence of hygromycin B (50 μM; Sigma-Aldrich, St. Louis, MO, USA, H3274) and puromycin (Sigma-Aldrich, P8833; 1 μg/mL for RNH1(D210N)-GFP and 0.4 μg/mL for RNH1(D210N)-BioID2-HA). Doxycycline (1 ng/mL; TAKARA BIO, Kusatsu, Japan, 631311) was added for 24 h to induce the expression of recombinant RNH1 and to downregulate the endogenous RNH1 expression by shRNA placed in the same vector. For cell synchronization in the G2 or prometaphases, cells were treated with the RO-3306 (9 μM; Sigma-Aldrich, SML0569) and nocodazole (100 ng/mL; Sigma-Aldrich, M1404), respectively. For transcription inhibition, the cells were released from nocodazole-mediated cell cycle arrest and treated with inhibitors such as actinomycin D (1 µg/mL; Sigma-Aldrich, A4262), cordycepin (50 µM; Sigma-Aldrich, C3394), α-amanitin (2 µg/mL; Sigma-Aldrich, A2263), triptolide (1 µM; Sigma-Aldrich, T3652), DRB (50 µM; Sigma-Aldrich, D1916), and roscovitine (50 µM; Sigma-aldrich, R7772) and with/without RAD51 inhibitor B02 (20 µM; Sigma-Aldrich, SML0364).

### 3.2. Generation of U-2-OS T-REx RNH1(D210N)-BioID2 Cell Line

We developed an RNase H1-based method for the identification of R-loop-associated proteins. We took advantage of the pAIO-based construct of catalytically inactive RNaseH1(D210N) fused with green fluorescence protein (GFP) [27]. A promiscuous biotin ligase termed BioID2 (Biotin IDentification 2) and HA epitope tag were introduced to the RNaseH1(D210N)-GFP construct in place of GFP. For this, the original RNaseH1(D210N)-GFP plasmid was cut with BamHI (Fermentas, Waltham, MA, USA), for which the recognition sites flanked the GFP tag. A plasmid containing BioID2-HA was kindly provided by Dr. Zdeněk Hodný. BamHI recognition sites were introduced on both ends of the BioID2-HA fragment by PCR. The sequences of the primers used for cloning are shown in Table S1. The resulting construct was transfected into U-2-OS T-REx cells, and clones expressing the RNaseH1(D210N)-BioID2-HA proteins were selected in the presence of hygromycin B. We observed that biotinylation occurs exclusively upon the addition of biotin into cell culture media and specifically in the cell nuclei, confirming proper targeting of the fusion protein.

### 3.3. Immunofluorescence Assay

Cells grown on autoclaved coverslips were transfected with siRNA and/or treated with drugs. After the treatment, the cells were permeabilized for 5 min with pre-extraction solution (25 mM Hepes, pH 7.7 (VWR international, RADNOR, PA, USA, 44148H); 50 mM NaCl (PENTA Chemicals, Prague, Czechia, 211207); 1 mM EDTA (AppliChem GmbH, Darmstadt, Germany, A3553); 3 mM MgCl_2_ (Sigma-Aldrich, M9272); 300 mM sucrose (Sigma-Aldrich, S8501); and 0.5% Triton X-100 (AppliChem GmbH, A4975)) on ice. After a brief wash, the cells were fixed with 4% paraformaldehyde (Sigma-Aldrich, F8775) for 15 min at RT. Fixation and all following incubations were performed in the dark. After fixation, the cells were blocked in 1% BSA/1× PBS for 10 min (BSA purchased from Sigma-Aldrich, A7030). The coverslips were then incubated with primary antibodies diluted in 1% BSA/1× PBS for 90 min at RT or overnight at 4 °C. The following antibodies and dilutions were used: anti-phospho histone H2A.X (Ser139) mouse monoclonal (Merck Millipore, Waltham, MA, USA, 05-636-AF647, 1:300), anti-53BP1 rabbit polyclonal (Santa Cruz Biotechnology, Inc., Dallas, TX, USA, sc-33760, 1:300), anti-Rad51 rabbit polyclonal (home-made), anti-nucleolin rabbit polyclonal (Abcam, Cambridge, United Kingdom, ab22758, 1:1000), and anti-DNA:RNA hybrid (S9.6) mouse monoclonal (Kerafast, Inc., Boston, MA, USA, ENH001, 1:200). The coverslips were washed 3 times with 1× PBS and then incubated with secondary antibodies diluted in 1% BSA/1× PBS for 30 min at RT, counterstained with 1 μg/mL 4′6-Diamidine-2′-phenylindole (DAPI) (Sigma-Aldrich, D9542) and mounted with Fluoromount-G mounting medium (Invitrogen, Carlsbad, CA, USA, 00-4958-02). The secondary antibodies and dilutions were Alexa Fluor 488 goat anti-rabbit IgG (Life Technologies, Carlsbad, CA, USA, A11034, 1:400), Alexa Fluor 488 goat anti-mouse IgG (Life Technologies, A11034, 1:400), Alexa Fluor 568 goat anti-rabbit (Life Technologies, A11036, 1:400), Alexa Fluor 568 goat anti-mouse (Life Technologies, A11031, 1:400), Alexa Fluor 647 goat anti-rabbit (Life Technologies, A21245, 1:400), and Alexa Fluor 647 goat anti-mouse (Invitrogen, A21235, 1:400). Representative images were acquired with a Leica DM6000 fluorescent microscope (63×/1.4 oil immersion). For the software-based analysis, automated image acquisition was performed on an IX83 microscope (Olympus, Tokyo, Japan) equipped with ScanR imaging platform using a 40×/1.3 NA oil objective or 60×/1.35 NA oil objective. Analysis of the acquired images, commonly referred to as a quantitative image-based cytometry (QIBC), was performed using ScanR Analysis software. The DAPI signal was used for segmentation of the images to identify individual nuclei. At least 800 cells were measured per condition.

### 3.4. Detection of RNA: DNA Hybrids with S9.6 Antibody

Staining of RNA:DNA hybrids with the anti-RNA:DNA hybrid antibody (S9.6) was performed using a previously published protocol [47]. Briefly, the cells grown on coverslips were fixed with ice-cold methanol for 10 min on ice and then permeabilized with acetone for 1 min on ice. After washing with 1× PBS and 4× saline-sodium citrate buffer (SSC) (0.6M NaCl; 60 mM sodium citrate (VWR international, L12557), the coverslips were blocked in 3% BSA/0.1% Tween-20/4× SSC (Tween-20 was purchased from Sigma-Aldrich, P7949) overnight on 4 °C. After a brief wash with blocking solution, the cells were incubated with S9.6 mouse monoclonal antibody and anti-nucleolin rabbit polyclonal diluted in a blocking solution overnight at 4 °C and 90 min at RT, respectively. After washing with 4× SSC, the coverslips were incubated with Alexa Fluor 488 goat anti-mouse IgG and Alexa Fluor 647 goat anti-rabbit IgG diluted in the blocking solution for 90 min, counterstained with DAPI (1 μg/mL), and mounted with Fluoromount-G mounting medium. The representative images were acquired with Leica DM6000 fluorescent microscope. For analysis of the S9.6 signal, the automated image acquisition was performed on an IX8 microscope (Olympus) equipped with ScanR imaging platform using a 60×/1.4 NA objective with oil immersion. The analysis of acquired images was performed using CellProfiler 4.0.7 (Broad Institute of MIT and Harvard, Cambridge, MA, USA) [48,49,50]. The DAPI signal was used for a segmentation of images to identify individual nuclei and for the determination of cell cycle phases. For each nuclear object, the nucleoplasmic signal of S9.6 was measured excluding the nucleolar S9.6 signal.

### 3.5. Small-Interfering RNA Transfections

A single siRNA against RAD51 (siRAD51; Microsynth AG, Balgach, Switzerland) or a predesigned pool of endoribonuclease-prepared siRNAs (esiRNA) against RAD51 (esiRad51; Sigma-Aldrich, EHU045521) was introduced to the cells at 40% cell confluency using Lipofectamine RNAiMAX (Thermo Fisher, 13778150) at a final concentration of 40 nM according to the instructions of manufacturer. RAD51 protein was depleted by transfection with siRNA or esiRNA for a total time of 36 h. Twelve hours after the transfection, the medium was exchanged with a fresh medium containing doxycycline (1 ng/mL) and nocodazole (100 ng/mL).

### 3.6. Sample Preparation for Flow Cytometry Measurement

For cell cycle measurement, the cells were trypsinized and collected into fresh medium. The cells were collected by centrifugation (250× g, 2 min, RT) and resuspended in PBS. For fixation, a cell suspension was added into a centrifuge tube with −20 °C ethanol (VWR, 02850) dropwise while vortexing and kept at −20 °C for at least 2 h. After washing with 1× PBS, the cells were resuspended in PBS containing 0.2 mg/mL RNase A (AppliChem GmbH, A3832) and incubated for 30 min at RT. Prior to measurement by flow cytometry, the propidium iodide solution (12.5 μg/mL propidium iodide (provided by IMG Flow Cytometry Facility), 0.1% Nonidet P-40 (AppliChem GmbH, A1694)) was added to the samples and measured by flow cytometer (LSR II, BD Biosciences, San Jose, CA, USA).

For detection of DNA replication, the cells were pulse-labelled with 20 µM 5-ethynyl-2′deoxyuridine (EdU; Thermo Fisher, A10044) for 3 h. EdU was added to nocodazole-released cells simultaneously with the RAD51 inhibitor (B02). The cells were then collected into fresh medium, washed, and resuspended in 1× PBS. The cells were then permeabilized for 5 min in pre-extraction solution (see the immunofluorescence assay) on ice and fixed with 4% PFA/1× PBS for 15 min at RT. After fixation, the cells were blocked in 1% BSA/1× PBS for 5 min at RT. The “click reaction” was used for detection of EdU incorporation. Briefly, after blocking, the cells were incubated in solution (100 mM Tris, pH 8.5 (Sigma-Aldrich, SML0364); 2 mM CuSO_4_ (Sigma-Aldrich, C2284); 100 mM ascorbate (Sigma-Aldrich, A7631); 5 nM Alexa Fluor Azide (Thermo Fisher, A10277)) for 30 min at RT. After washing, the cells were resuspended in PBS containing 0.2 mg/mL RNase A for 30 min. Prior to measurement by flow cytometer, the cells were counterstained with DAPI, washed, resuspended in 1× PBS, and measured by flow cytometer (LSR II, BD Biosciences).

### 3.7. Preparation of Cell Extracts and Western Blot Analysis

Harvested cells were permeabilized with pre-extraction solution (see the immunofluorescence assay) for 5 min on ice. The cells were then scraped in 2× SDS-SB lysis buffer (125 mM Tris, pH 6.8; 4% (*w*/*v)* SDS ( SERVA Electrophoresis GmbH, Heidelberg, Germany, 20783); 20% (*v*/*v*) glycerol (Sigma-Aldrich, G7757)) into a 1.5 mL microcentrifuge tube and incubated on 95 °C for 10 min. The cell lysates were sonicated and centrifuged (16,000× *g* for 10 min at RT), and protein concentration was measured by BCA assay and adjusted to same level; 100 mM of DTT (Sigma-Aldrich, D0632) and 0.01% bromophenol blue were added to the cell lysates, and 20–30 μg of total protein from cell lysates was loaded onto 10–12% SDS-PAGE gels. After electrophoretic separation, the proteins were transferred onto a nitrocellulose (VWR international, 10600003) or methanol-preactivated PVDF membrane (VWR international, 10600021) in a wet-transfer apparatus in transfer buffer (10% methanol (P-lab, M 03103); 2.5 mM Tris; 19.2 mM glycine (AppliChem GmbH, A1377)) at 300 mA for 1.5 h at 4 °C. After transfer, the membrane was blocked in 1% BSA for 30 min at RT while gentle agitation and incubated with primary antibody was diluted in 1% BSA/TBS-T (20 mM Tris-HCl (AppliChem GmbH, A1086), 150 mM NaCl, 0.1% Tween-20) O/N at 4 °C on a roller. The primary antibodies used were anti-RAD51 (rabbit, home-made), anti-RNH1 (rabbit, home-made), anti-TFIIH (p89/XPB) (sc-293; Santa Cruz Biotechnology), and anti-RNAPII (clone 7C2, a gift from J.-M. Egly). The membrane was then washed with TBS-T solution and incubated with horseradish peroxidase-coupled secondary antibody diluted in 1% BSA/TBS-T for 60 min at RT. The secondary antibodies used were goat anti-mouse IgG-HRP (Sigma-Aldrich, A4416) and goat anti-rabbit IgG-HRP (Sigma-Aldrich, A0545). Afterward, the membrane was washed with TBS-T and the protein bands detected a luminol-based reaction using a chemiluminescence reagent (Pierce ECL Western Blotting Substrate, Thermo Fisher, 32209).

### 3.8. Chromatin Immunoprecipitation and qPCR

ChIP assay was performed using a ChIP-IT Express kit (Active Motif, Inc., Carlsbad, CA, USA) according to the manufacturer’s instructions. Briefly, U-2-OS T-REx RNH1(D210N)-GFP cells were synchronized in mitosis by treatment with nocodazole for 24 h. Simultaneously, the induction of RNH1(D210N)-GFP expression was induced by the addition of doxycycline to a final concentration of 1 ng/mL. After 24 h, mitotic cells were isolated by mitotic shake-off, fresh medium with or without 20 µM B02 was added, and the cell were then released for 3 h. The cells were then permeabilized with pre-extraction solution (see the immunofluorescence assay) and fixed with 1% formaldehyde for 15 min while gently agitating. Cross-linked chromatin was sheared by sonication (Bioruptor, Diagenode, Liège, Belgium). Fragmented chromatin was immunoprecipitated with anti-GFP antibody (Abcam, ab290) overnight at 4 °C. After elution, reversal of the cross-links, and proteinase K digestion (Sigma-Aldrich, 3115852001), the DNA was purified using QIAquick PCR purification kit (Qiagen, Hilden, Germany, 28104) and analyzed by qPCR. The sequences of primers are showed in Table S1. The data were analyzed by taking the cycle threshold values from the qPCR assay. Relative enrichment was calculated as the amount of precipitated DNA relative to the enrichment of the amount of DNA in input chromatin and normalized to the amplicon H27 of our DMSO-treated control sample. To confirm the quality of the treatment, the intensity of GFP signal in harvested cells was analyzed by flow cytometry.

### 3.9. Immunoprecipitation of Biotinylated Proteins

U-2-OS T-REx RNH1(D210N)-BioID-HA cells were treated with doxycycline (1 ng/mL) for 24 h to induce the expression of RNase H1(D210N)-BioID-HA. During the treatment, cells were treated with nocodazole (100 ng/mL) for the last 16 h. The following day, the mitotic cells were shaken off by gentle agitation. The mitotic cells were then washed with fresh pre-warmed medium and resuspended in pre-warmed medium containing 20 μM B02 and 50 μM biotin (Sigma-Aldrich, B4501). The cells were released from nocodazole-induced cell cycle arrest for 4 h.

For the immunoprecipitation (IP) experiments, the cells were trypsinized, collected by centrifugation (250× *g*), and washed with ice-cold 1× PBS. In order to obtain the nuclei, the cells were resuspended in a fractionation buffer (10 mM Hepes-NaOH, pH 7.9; 10 mM KCl (Sigma-Aldrich, P9333); 1.5 mM MgCl_2_; 0.34 M sucrose; 10% glycerol; and 1 mM DTT) supplemented with 1 mg/mL digitonin (Sigma-Aldrich, D141) and incubated on ice for 5 min. After centrifugation (1500× g, 10 min, 4 °C), the cells were gently washed with fractionation buffer without disrupting the pellet, resuspended in lysis buffer (50 mM Tris-HCl, pH 7.5; 120 mM NaCl; 0.5% Nonidet P-40) supplemented with protease inhibitor cocktail (Roche, 11,873,580,001), and incubated in lysis buffer for 5 min on ice. After sonication and brief centrifugation (16,000× *g*, 10 min, 4 °C), the supernatants were incubated with Dynabeads MyOne Streptavidin C1 (Thermo Fisher, 65002) for 30 min at RT. The beads were then washed with the following sequence of solutions: (1) 2% SDS; (2) 0.1% sodium deoxycholate (Sigma-Aldrich, D6750), 1% Triton X-100, 1 mM EDTA, 500 mM NaCl, 50 mM Hepes, pH7.5; (3) 0.5% sodium deoxycholate, 0.5% Nonidet P-40, 1 mM EDTA, 250 mM LiCl (Sigma-Aldrich, L9650), 10 mM Tris-HCl, pH 7.5; and (4) 50 mM Tris-HCl, pH 7.5, 120 mM NaCl. Seventy-five percent of the beads from every sample were subjected to snap-freezing in liquid nitrogen and sent for mass spectrometry analysis. The remaining 25% of beads were incubated at 95 °C in 2× Laemli sample buffer for 5 min, and the eluted proteins were then analyzed by Western blotting.

### 3.10. Protein Digestion

Protein digestion was performed in the Laboratory of Mass Spectrometry [Biotechnology and Biomedicine Centre of the Academy of Sciences and Charles University, Vestec, Czechia (Biocev)] as follows. Immunoprecipitated samples were resuspended in 100 mM TEAB containing 2% SDC. Cysteines were reduced with 5 mM final concentration of TCEP (60 °C for 60 min) and blocked with 10 mM final concentration of MMTS (10 min, RT). The samples were cleaved on beads with 1 µg of trypsin at 37 °C O/N. After digestion, the samples were centrifuged and the supernatants were collected and acidified with TFA to 1% final concentration. SDC was removed by extraction to ethylacetate [51]. The peptides were desalted using in-house-made stage tips packed with C18 disks (Empore) according to Rappsilber et al. [52].

### 3.11. nLC-MS 2 Analysis

MS 2 analysis was performed in the Laboratory of Mass Spectrometry (Biocev) as follows. Nano Reversed phase column (EASY-Spray column, 50 cm *×* 75 µm ID, PepMap C18, 2 µm particles, 100 Å pore size) was used for LC/MS analysis. Mobile phase buffer A was composed of water and 0.1% formic acid. Mobile phase B was composed of acetonitrile and 0.1% formic acid. The samples were loaded onto the trap column (Acclaim PepMap300, C18, 5 µm, 300 Å Wide Pore, 300 µm *×* 5 mm, 5 Cartridges) for 4 min at 15 μL/min. The loading buffer was composed of water, 2% acetonitrile, and 0.1% trifluoroacetic acid. The peptides were eluted with a Mobile phase B gradient from 4% to 35% B in 60 min. Eluting peptide cations were converted to gas-phase ions by electrospray ionization and analyzed on a Thermo Orbitrap Fusion (Q-OT-qIT, Thermo Fisher). Survey scans of peptide precursors from 350 to 1400 *m*/*z* were performed at 120K resolution (at 200 *m*/*z*) with a 5 × 10^5^ ion count target. Tandem MS was performed by isolation at 1.5 Th with the quadrupole, higher-energy collisional dissociation (HCD)-type fragmentation with a normalized collision energy of 30, and rapid scan MS analysis in the ion trap. The MS2 ion count target was set to 10^4^, and the max injection time was 35 ms. Only those precursors with charge states 2–6 were sampled for MS2. The dynamic exclusion duration was set to 45 s, with a 10 ppm tolerance around the selected precursor and its isotopes. Monoisotopic precursor selection was turned on. The instrument was run in top speed mode with 2 s cycles [53].

### 3.12. Data Analysis

The initial data analysis and quantification was performed in the Laboratory of Mass Spectrometry (Biocev). All data were analyzed and quantified with the MaxQuant software (version 1.6.1.0, Max Planck Institute of Biochemistry, Planegg, Germany) [54]. The false discovery rate (FDR) was set to 1% for both proteins and peptides, and we specified a minimum length of seven amino acids. The Andromeda search engine was used for the MS/MS spectra search against the *Human* database (downloaded from Uniprot on September 2017, containing 20,142 entries). Enzyme specificity was set as the C-terminal to Arg and Lys, also allowing for cleavage at the proline bonds and a maximum of two missed cleavages. Dithiomethylation of cysteine was selected as fixed modification, and N-terminal protein acetylation and methionine oxidation were selected as variable modifications. The “match between runs” feature of MaxQuant was used to transfer identifications to other LC-MS/MS runs based on their masses and retention time (maximum deviation 0.7 min), and this was also used in the quantification experiments. Quantifications were performed with the label-free algorithms described recently. Data analysis was performed using Perseus software (version 1.6.0.7, Max Planck Institute of Biochemistry, Planegg, Germany) [55]. Three independent experiments were performed.

### 3.13. Statistics

All statistical analysis was performed using Graph Pad prism software (version 5.04, San Diego, CA, USA). Student’s t-test was used throughout for statistical comparisons. The *p*-values are indicated as follows: * *p* < 0.05, ** *p* < 0.01, *** *p* < 0.005, and **** *p* < 0.001.

## Data Availability

The data presented in this study are available on request from the corresponding author.

## Supplementary Materials

The following are available online at https://www.mdpi.com/article/10.3390/ijms22073740/s1.
