# Supplementary material for: RAD51 Inhibition Induces R-Loop Formation in Early G1 Phase of the Cell Cycle
Source: Int J Mol Sci. 2021 Apr 3;22(7):3740. doi: 10.3390/ijms22073740 (PMC8038378; doi:10.3390/ijms22073740)
Supplement: Supplementary file 1 [file ijms-22-03740-s001.pdf]

Supplementary Information

RAD51 inhibition induces R-loop formation in early G1 phase of the cell cycle

Zuzana Nascakova[1,2], Barbora Boleslavska[1,2], Vaclav Urban[1], Anna Oravetzova[1,2], Edita Vlachova[1], Pavel Janscak[1,3#] & Jana Dobrovolna[1#]

[1]Institute of Molecular Genetics, Academy of Sciences of the Czech Republic, 142 20, Prague, Czech Republic

[2]Faculty of Science, Charles University in Prague, 128 00, Prague, Czech Republic

[3]Institute of Molecular Cancer Research, University of Zurich, 8057 Zurich, Switzerland

# corresponding author

email: jana.dobrovolna@img.cas.cz, pjanscak@imcr.uzh.ch

Table S1. The sequences of primers used for qPCR

Amplicon	Forward	Reverse
H42	AGAGGGGCTGCGTTTTCGGCC	CGAGACAGATCCGGCTGGCAG
H42.9	CCCGGGGGAGGTATATCTTT	CCAACCTCTCCGACGACA
H0.1	TCTGGCGACCTGTCGTCGGA	CTCGGACGCGCGAGAGAACAG
H0.4	CAGGCGTTCTCGTCTCCG	CACCACATCGATCGAAGAGC
H4	CGACGACCCATTCGAACGTCT	CTCTCCGGAATCGAACCCTGA
H6	CAGCTAGCTGCGAGAATTAATG	CGATTGATCGGCAAGCGAC
H8	AGTCGGGTTGCTTGGGAATGC	CCCTTACGGTACTTGTTGACT
H11	GGACCAGGGGAATCCGAC	CGCTTCATTGAATTTCTTCAC
H13	ACCTGGCGCTAAACCATTCGT	GGACAAACCCTTGTGTCGAGG
H18	GTTGACGTACAGGGTGGACTG	GGAAGTTGTCTTCACGCCTGA
H27	CCTTCCACGAGAGTGAGAAGCG	CTCGACCTCCCGAAATCGTACA

MCS-BamH1_forward	TC**GGATCC**ACTTCAAGAACCTGATCTGGC
MCS-BamH1_reverse	AGC**GGATCC**CTATGCGTAATCCGGTACATC

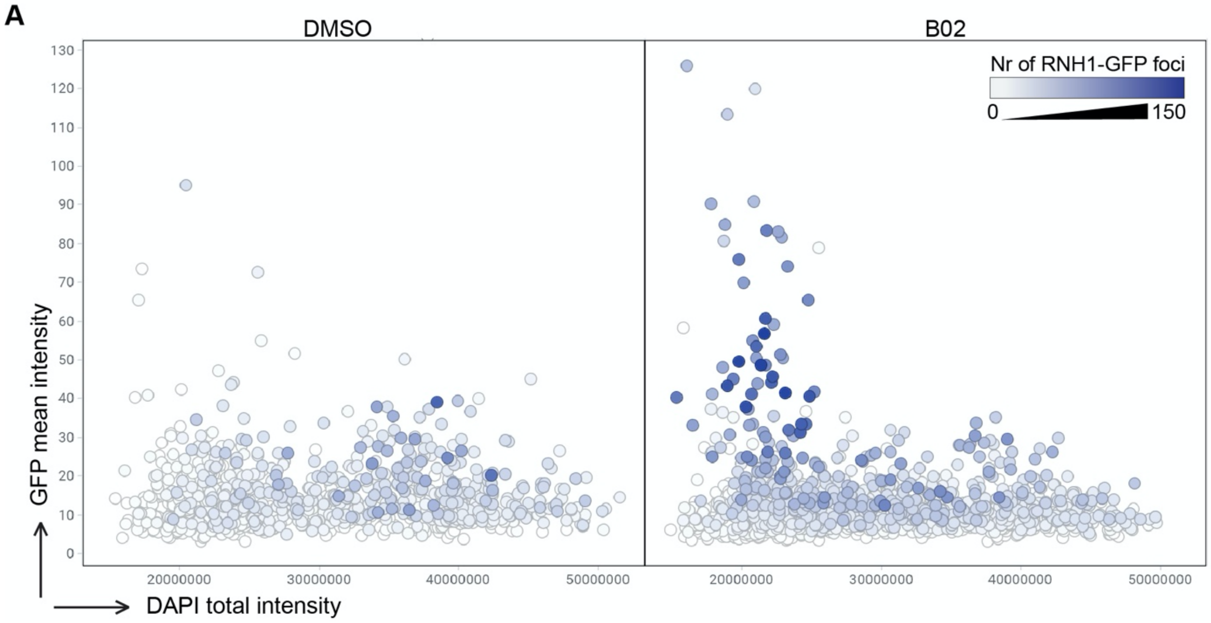

Figure S1. B02-induced R-loop formation in asynchronous cell population

(**A**) U-2-OS T-Rex [RNH1(D210N)-GFP] cells were treated with doxycycline (1 ng/ml) for 24 h. B02 (20 μM) was added for the last 6 h of doxycycline treatment. Cell were pre-extracted before fixation, counterstained with DAPI and subjected to image-based quantification of GFP intensity and number of GFP foci per cell. The cell cycle distribution was evaluated based on the DAPI intensity and is shown on x axis and GFP mean intensity is shown on y axis. The number of RNH1(D210N)-GFP foci per cell is increasing with an increasing intensity of blue color.

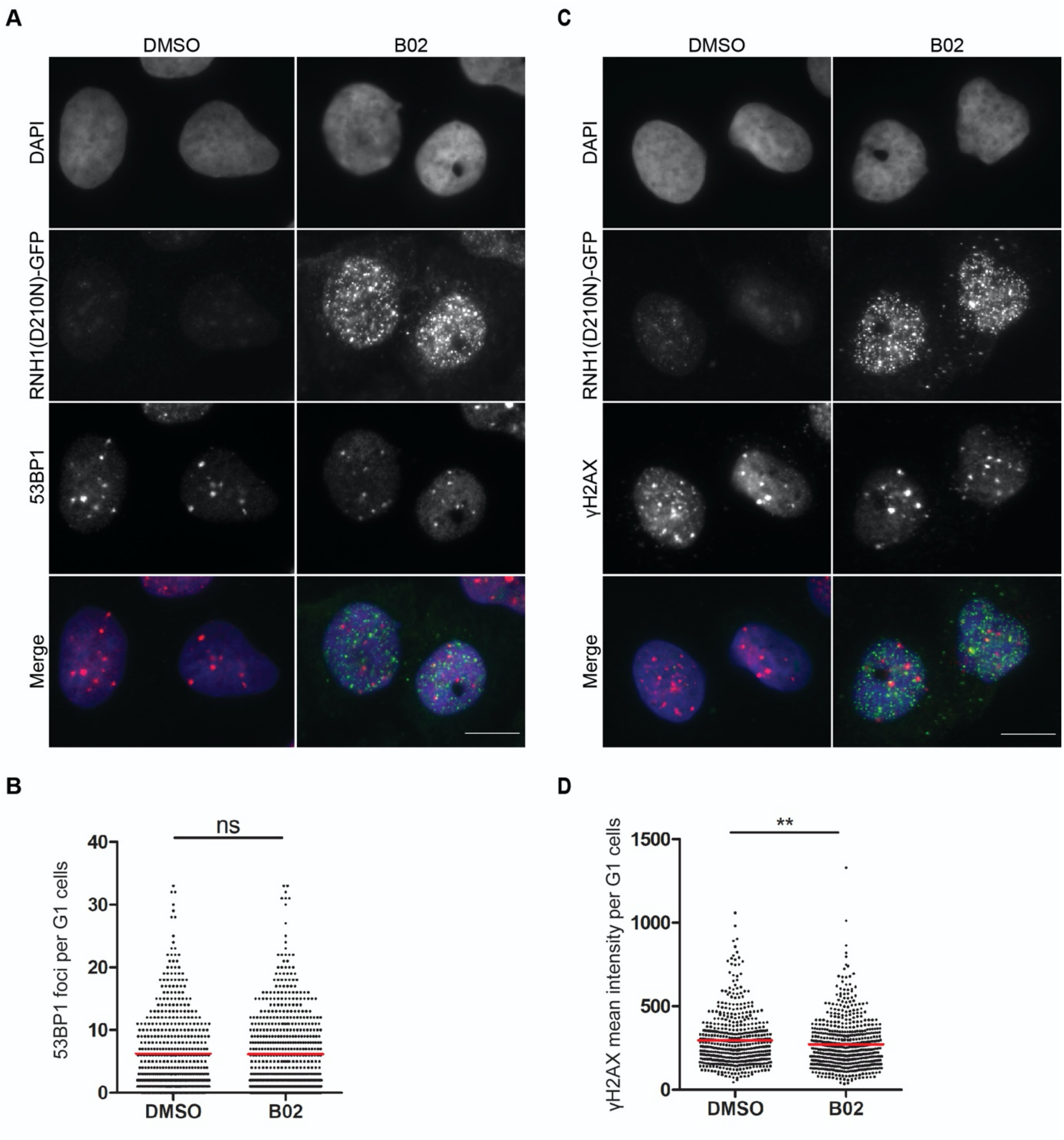

Figure S2. DNA damage markers upon B02 treatment

(**A-D**) U-2-OS T-Rex [RNH1(D210N)-GFP] cells were treated with doxycycline (1 ng/ml) for 24 h, and nocodazole (100 ng/ml) for the last 20 h of doxycycline treatment. B02 (20 μM) was added for 3 h post-release. Cells were pre-extracted before fixation and subjected to immunostaining of DNA damage markers including 53BP1 and γH2AX. Representative images are shown in (**A**) and (**C**) alongside with the image-based quantification of number of 53BP1 foci (**B**) and γH2AX intensity (**D**) per G1 cell. Scale bar in (**A**) and (**C**) represents 10 μm. Data in (**B**) and (**D**) are pooled from 3 individual experiments. Statistical significance was determined using Mann-Whitney test (**** $p<0.0001$, *** $p<0.005$, ** $p<0.01$, * $p<0.05$, ns, not significant).

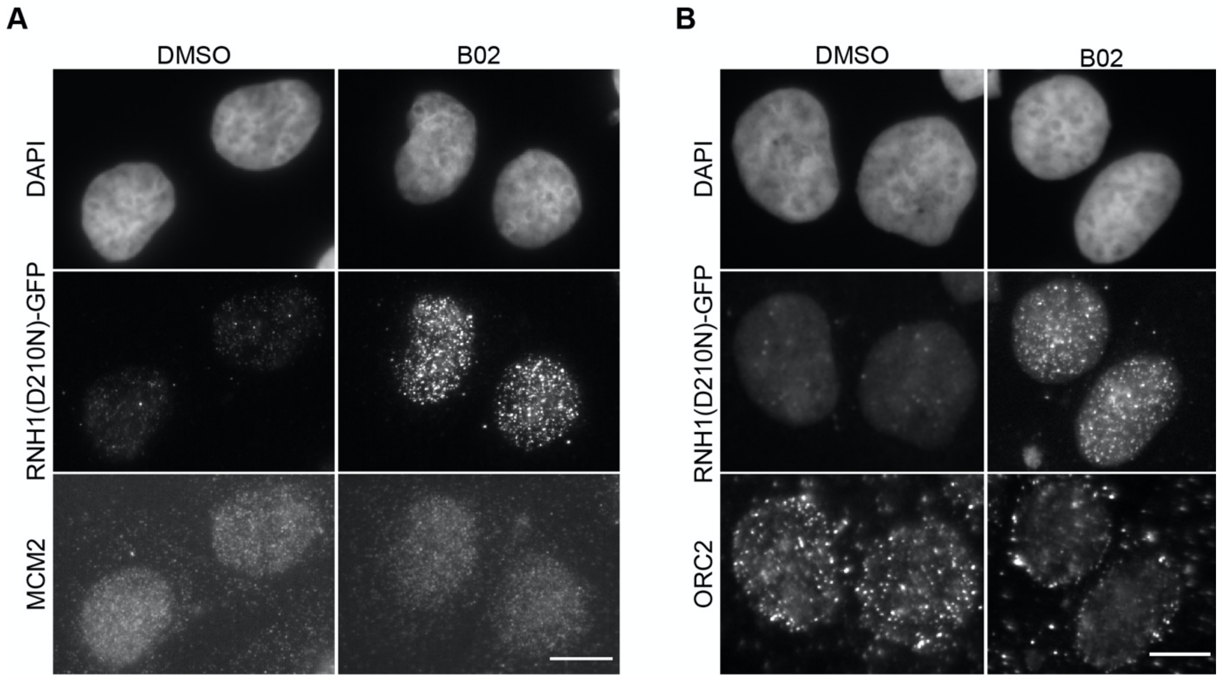

Figure S3. Staining of replication origin licensing proteins

(**A-B**) U-2-OS T-Rex [RNH1(D210N)-GFP] cells were treated with doxycycline (1 ng/ml) for 24 h, and nocodazole (100 ng/ml) for the last 20 h of doxycycline treatment. B02 (20 μM) was added for 3 h post-release. Cells were pre-extracted before fixation and subjected to immunostaining of MCM2 in (**A**) or ORC2 in (**B**). Scale bar represents 10 μm.
